# Challenges faced by adolescent girls on menstrual hygiene management: School-based study, Siha, Kilimanjaro, Tanzania

**DOI:** 10.1371/journal.pgph.0002842

**Published:** 2024-06-13

**Authors:** Andrew Method, Johari Hassan, Odilia Assenga, Placidia Kamugisha, Theresia Kawishe, Frank Luchagura, Peter Msaka, Milka Singu, Deogratius Bintabara

**Affiliations:** 1 City Medical Officer of Health of Dodoma City Council, Formerly District Medical Officer, Siha District Council in Kilimanjaro Region, Kilimanjaro, Tanzania; 2 Council Health Management Team (CHMT)–Siha District Council in Kilimanjaro, Tanzania; 3 Department of Community Medicine, The University of Dodoma, Dodoma, Tanzania; University of Ghana, GHANA

## Abstract

Menstrual hygiene management (MHM) has received increasing attention as a public health issue globally. Governments and stakeholders have started to engage communities to address barriers and challenges faced by adolescents in and out of school. This study, conducted in Siha District, northern Tanzania, responds to the call for evidence sensitive to local barriers and challenges to inform successful strategies in MHM. Institutional-based cross-sectional study which involved 400 school girls aged 10 to 19 years old who attained menarche were randomly selected in four secondary and advanced level government schools from September 2019 to January 2020. Bivariate and multivariable logistic regression analysis were employed. A P-value less than 0.05 was used to declare statistical significance. Among all the girls who participated in the study 30% reported missing school due to menstruation while 56% of the girls reported using toilets as changing places at school. The use of reusable sanitary material was 52% compared with non-reusable materials which was 48%. In urban areas, 34.5% of students reported missing school due to menstruation compared to 25% who reported in rural areas. The findings show that school absenteeism among adolescent girls during menstruation is significantly associated with a headache (Adjusted odds ratio (AOR) = 3.3 (95% CI:1.32–8.23)) and abdominal waist pain (AOR = 8.50 (95% CI: 6.27–15.56)), lack of changing rooms in school (AOR = 5.85 (95% CI: 4.82–7.93)). In addition, the high cost of sanitary pads was mentioned as one of the main reasons for students not using sanitary pads. This study calls for promoting MHM-friendly practices in schools to create a supportive and conducive learning environment for adolescent girls. Ongoing infrastructure improvements such as the construction of classrooms and toilets in schools should include the construction of proper changing places to reduce the number of adolescent girls who miss school due to menstruation.

## 1. Introduction

Menstruation is a natural part of the reproductive cycle during which blood is lost through the vagina [1, 2] Most adolescents experience their first menstruation (menarche) between the ages of 11 and 14 years [2]. It is a developmental milestone and is a transition period for girls from childhood to womanhood [3]. However, some girls start as early as 8 and some at 17 or older [2]. Hygiene during the period of menstruation is fundamental to the dignity and well-being of women and girls and an issue that every girl has to deal with once she enters adolescence around the age of 12 and until she reaches menopause [1, 2].

Menstrual hygiene products such as tampons, sanitary pads, menstrual cups, cloths, paper material, or plant material are used by women and girls to absorb menstrual blood to maintain personal hygiene during the period of menstruation and to prevent physical discomfort and leakages [1, 2]

WHO and UNICEF in 2012, defined MHM as: ’Women and adolescent girls using a clean menstrual management material to absorb or collect blood that can be changed in privacy as often as necessary for the duration of the menstruation period, using soap and water for washing the body as required and having access to facilities to dispose of used menstrual management materials [1, 2, 4]. Safe and effective menstrual hygiene management is a trigger for better development for adolescent girls, equipping them with knowledge and skills on MHM, which enhance their self-esteem, and positively impact their academic performance [1, 3].

Menstrual hygiene management (MHM) has received increasing attention as a public health issue [1, 2]. After a history of silence, stakeholders from governments to local charities have started to speak about the barrier that the management of menstruation presents to gender equality and the potential for programs to address the problem. Even though menstruation is a natural process, it is linked with several misconceptions and malpractices which may result in adverse health outcomes. Poor hygiene during menstruation has been associated with serious ill health, including reproductive tract and urinary tract infections [5].

Adolescents (10–19 years) make up 16% of the world population and 23% of the Sub-Saharan African population [1]. An adolescent is at a critical time of rapid physical, psychological, and cognitive changes that affect how they feel, think, make decisions, and interact with the world around them [3]. Globally, according to a World Health Organization (WHO) report, 2.3 billion girls and women didn’t manage their menstruation safely due to a lack of facilities for MHM, high cost, and ignorance [4]. As a result, their option is to use old clothes, or other unhygienic materials as menstrual absorbents, which may expose them to infections and other related health consequences [4].

Menstrual hygiene-related problems have negative impacts on girls’ lifestyle, health, and developmental opportunities including absence from their school. Girls may also become worried due to consequences of poor MHM including, offensive smell, symptoms of reproductive tract infection such as itching of the vulva, pain during urination, and vaginal discharge [3]. The barriers to menstrual hygiene management faced by adolescent schoolgirls in low-income countries are gaining interest at practice and policy levels as they face many challenges [1]. The challenges include inadequate water, sanitation, and disposal facilities for the management of menses with privacy and dignity and insufficient guidance to help girls feel confident in attending school during menses [6]. The effective management of menstruation has been identified as an under-recognized challenge for girls in low-income contexts [7].

According to estimates of the United Nations Children’s Fund (UNICEF), about 10% of school-age African girls didn’t attend school during menstruation or dropped out at puberty due to a lack of cleanliness and separate toilet facilities for female students at schools [1, 2, 8]. Studies in Africa revealed that 50% up to 70% of girls missed on average 1.6–2.1 days from school every month due to menstrual-related issues such as shame, pain, and uncomfortable among others [9]. In Ethiopia, about 43% up to 51% of students missed their class during the menstruation period. In Uganda, 9.5% of girls said that in general, they didn’t attend school during menstruation, and 17.3% gave menstruation as the main reason for them missing school (29.3% of those who missed at least 1 day in the last month) [8, 10, 11].

The most commonly reported reasons for missing school during menstruation were stomach or back pain (92.5%), feeling generally unwell (60.0%), fear of leaking blood (38.5%), and lack of privacy for changing (38.5%). The majority said pain was the main reason for missing school (85.7%) [10]. In another study of the 149 school girls involved in the study, 24.8% were absent from school or classrooms at least once because of a lack of any of the MHM facilities at their schools [8].

Studies have shown girls lack water, soap, privacy, and space to change; adequate time to manage their menses comfortably, safely, and with dignity; and hygienic sanitary products and sometimes underwear [12–14]. Few girls reported that parents had purchased pads, but most did so inconsistently. Unreliable absorbents kept girls from school, particularly where girls were using old or found clothes. Girls with no absorbents were absent. Most girls reported knowing others who struggled to find absorbents and thus stayed home [15].

In a study done in northern Tanzania 47% of girls left school early during their last period, 31% did not participate in class as much as normal, and 33% concentrated less while in school [16]. Fear and shame, alongside cramps and pain, are commonly reported reasons for absenteeism or lower participation and concentration in the classroom [16]. Studies have noted poor sanitation in schools and lack of access to good quality sanitary products can be associated with lower enrolment in schools, absenteeism, and dropout [17].

In Kilimanjaro, Tanzania, girls expressed frustration with managing menses en route to and in school [18]. A lack of adequate guidance, facilities, and materials for girls to manage their menstruation in school is a neglected public health, social, and educational issue that requires prioritization, coordination, and investment [14].

Menstrual hygiene practices are affected by cultural norms, parental influence, personal preferences, economic status, and socioeconomic pressures. Menstrual beliefs refer to misconceptions and attitudes towards menstruation within a given culture or religion. Menstrual beliefs, knowledge, and practices were all interrelated to menstrual hygiene management, girls were not adequately informed about the realities of menstruation [19]. Even touching menstruating women was considered toxic, they were prohibited from cooking and taking certain foods, and not allowed to participate in religious activities or to contact religious articles [19].

In Tanzania, 66% of boys reported that menstruating women and girls in their homes are restricted from daily activities, most commonly cooking (50% of restrictionist households) Touching water sources or animals, washing dishes, and attending public gatherings are restricted in roughly 30% of restrictionist households [16]. Studies on menstrual hygiene management (MHM) have shown causal links between poor MHM and school absenteeism [13]. Girls have indicated receiving inadequate guidance before their first menstrual period and experiencing fear, shame, and embarrassment in managing menstruation, particularly while in school [14, 20, 21]. They mentioned health workers (80%) and school curricula (76%) as their main sources of information on menstruation [16].

The Tanzanian government has been quite proactive in addressing sexual and reproductive health needs for young people, creating policies like the National Multisectoral and Strategic Framework (NMSF) on HIV and AIDS and the National Adolescent Sexual and Reproductive Health Strategy (ASRH) [16]. An increasing number of schools and pupils in recent years, particularly in community schools however was not accompanied by an increase in sanitary facilities, including menstruation facilities. This is partly because, until recently, MHM has been largely overlooked by the Water, Sanitation, and Hygiene (WASH) sector in general, and Tanzania in particular [8].

This study, conducted in the District of Siha, northern Tanzania, responds to the call for evidence sensitive to local barriers and challenges to MHM with the understanding that not all results will be generalizable to Tanzania nationally. Local District Councils have been charged by national and regional governments with the task of researching public/population health to identify community-level needs to inform community interventions and ultimately, national policy. This study, under the auspices of the District Health Department, was conducted by the Council Health Management Team (CHMT, the designated public health/community health team and The District Medical Officer (DMO) led the project as a response to the President’s Office Local Government Authority to the District Council directives to the use of evidence-based data in making decision and train members of the Council Health Management Team (CHMT) in research methods.

## 2. Methods

### 2.1. Study design and area

This study was a cross-sectional study using developed survey questionnaires, assessing MHM knowledge, practice, and challenges. Participants were recruited from Sept 2019 to January 2020. They were given self-administered questionnaires which they filled out without the presence of a teacher and were able to ask questions for clarifications from research assistants. This study was conducted in Siha District which has a total population of 139,019 of which 67,213 are males and 71,729 are females [22, 23]. This District has an annual growth rate of 2.8 and a population density of 88 people per sq km [23]

### 2.2. Study population

Adolescent female students aged 10 to 19 years old who attained menarche were randomly selected in four ordinary and advanced level government schools out of 15 ordinary and 7 advanced level schools in the district.

### 2.3. Exclusion criteria

Adolescent girls not willing to be part of the study were excluded.

#### 2.3.1. Sampling and sample size

A multistage cluster sampling technique was performed in the Kilimanjaro region and Siha District was selected. The sample size was calculated using $\text{n}=\left\{\frac{z^{2}p[1-p]}{e^{2}}\right\}$ Where: n is the population size, z is the z-score, e is the margin of error, and p is the standard of deviation hence sample size was calculated with a 95% confidence level whereby students selected from four secondary schools participated and the design effect was 2 and 0.05 allowed for the margin of error. P was 17.3% which was a prevalence in a study done in Uganda [10].

Therefore, the Sample size calculated by $\text{n}=\left\{\frac{1.96^{2}17.3[100-17.3]}{5^{2}}\right\}*2=440$

#### 2.3.2. Note

As per the above calculation, the exact sample size population for this study is 220. However, to make more representative sampling and minimize high errors in the filling of close-ended questionnaires by respondents, it was multiplied by two to obtain optimum results from the larger number of respondents i.e. 220 * 2 = 440. To significantly minimize sampling error, the sample size was adjusted to 400 participants.

### 2.4. School sampling and participant selection

Four government schools were purposefully selected based on geographical location to include two rural and two peri-urban schools. All schools have day and boarding students and advanced-level classes.

In each school the class teachers (one teacher appointed by the school for each class) and research assistants checked the class registry to identify adolescents between the ages of 10 to 19 years old in each school who had reached menarche by systematic random sampling in which a fixed starting point from attendance register was chosen and the following participant name was obtained, they were assigned even numbers in the registry then identified by using those numbers until 100 students were obtained in each school. Only selected girls of these classes who attended the school on the day of study and were willing to participate in the study were included. Girls from these classes who had not yet had menarche were excluded. The research assistants explained the purpose of the study to all participants. The research assistant obtained oral and written consent from all participants.

### 2.5. Pilot questionnaire

Questionnaires for students were developed in English and then translated into Swahili. The Swahili version was discussed by the Community Health Management Team (CHMT) to reach a consensus on the selection of the appropriate terms to be used. The pilot testing of student and key informant questionnaires was done at Magadini (Peri-urban) and Oshara Secondary School (rural) of which 60 students participated in the pilot, the schools that participated in the pilot were not included in the study. Results were tabulated, checked and questions adjusted for clarity. The pilot process also allowed CHMT to investigate and be sensitized to school settings including latrines for cleanness and safety, water and soap access, and availability of a changing area.

### 2.6. Data collection

Data collection was conducted through pre-tested Swahili questionnaires containing semi-structured questions administered by a CHMT researcher/ research assistant. Pilot questionnaires were coded and analyzed using SPSS version 20. Data collection was completed in 2020. Final data analysis was completed in 2022 using STATA version 16.

The self-administered questionnaire was given to girls in either classrooms, offices, or in some schools under the tree in the absence of male students or teachers. Female members of the research team were briefed on the purpose of the study and the method of completing the questionnaire. Voluntary willingness from the respondents to participate in the study was sought before they were requested to fill out the questionnaires. girls were allowed to enquire about any clarifications from the research team.

### 2.7. Data processing and analysis

All questionnaires were assigned serial numbers for participant anonymity. The results were translated into English. Data coding for the variables to be measured was done initially using SPSS version 20. Final data cleaning and analysis including all necessary biostatistics computations was completed using STATA version 16. Both descriptive and analytical statistics were computed. Bivariate and multivariate models were run to assess any relationship between each independent variable (socio-demographic factors, school environment, parental or family factors, knowledge about menstruation and its hygienic management and disposable sanitary pad use) and outcome variables (disposable sanitary pads use and absenteeism from school). Crude and adjusted odds ratios were used to ascertain effect sizes for any association between the dependent and predictor variables while significance was determined using 95% confidence intervals. Independent variables found to be significant with a p-value less than 0.05 at the bivariate level were included in a multivariate logistic regression model for the dependent variable to control potential confounding variables.

### 2.8. Variables definition

#### 2.8.1. Menstrual hygiene management

The study adopted the menstrual hygiene management definition proposed by the World Health Organization and UNICEF as the "use of clean menstrual management material to absorb or collect menstrual blood that can be changed in privacy as often as necessary for the duration of a menstrual period, using soap and water for washing the body as required and having access to safe and convenient facilities to dispose of used menstrual management materials" [1].

#### 2.8.2. Good menstrual hygiene management practice

Girls self-reported MHM practices through structured questions. A girl was considered as having good menstrual hygiene management practice, if she scored mean or above the mean from practice-related questions, otherwise considered as having poor menstrual hygiene management practice. Each girl was asked nine practice-related questions concerning MHM and each correct answer was given a value of “1” if she answered correctly and a value of “0” otherwise.

Absorbents were considered clean if they were new cloth or sanitary pads, with old cloth and other items such as toilet paper, mattress, sponge, or underwear alone considered inadequate. Changing absorbents three or more times per 24 hours was required to be considered adequate MHM.

#### 2.8.3. Knowledge of menstrual hygiene

A girl was considered knowledgeable on MHM if she scored mean or above the mean from knowledge-related questions. Each girl was asked 7 knowledge-related questions concerning MHM and each correct answer was given a value of “1” if she answered correctly and a value of “0” otherwise.

#### 2.8.4. Menstrual-related school absenteeism

This was defined as missing one or more days of school because of menstruation or its management.

### 2.9. Ethical considerations

Ethical clearance was obtained from the National Institute for Medical Research -Tanzania (NIMR) after they reviewed the proposal. Local permission was obtained from the Siha District Executive Director and District Administrative Secretary and all logistic arrangements for the study were made in close consultation with relevant authorities. Written informed consent was obtained from each participant before enrolment into the study and study procedures and objectives were explained to them by the research assistants. In addition, written consent was obtained from parents on behalf of participants below 18 years. Individual participants were assured of their freedom to participate and to drop out of the study at any time without any consequence. All study participants were assured of the confidentiality of responses and information provided. The whole research process adhered to the principle of anonymity by redacting the personally identifiable information of the participants on the questionnaires.

## 3. Results

### 3.1. Socio-demographic characteristics

A total of 400 eligible girls participated in the study (100 from each school). More than 80% of the students who participated in the study were above 15 years hence are in middle to late adolescence. It was noted that 74.75% of girls spent more than 20 minutes from home to school and 58% of girl’s mothers had primary-level education (Table 1).

**Table 1:** Baseline characteristics of the respondents (N=400).

Variable	Frequencies	Percentage
**Age**		
13–14	46	11.50
15–16	125	31.25
17–19	229	57.25
**Education level**		
Form One	64	16.00
Form Two	69	17.25
Form three	131	32.75
Form Four	102	25.50
Form Five	17	4.25
Form Six	17	4.25
**Ethnicity**		
Masai	84	21.00
Meru	78	19.50
Chagga	124	31.00
Others	114	28.50
**Religion**		
Christians	288	72.00
Muslims	105	26.25
Others	7	1.75
**Mother’s education Level**		
Primary Education	232	58.00
Secondary Education	79	19.75
College Education	18	4.50
University Education	6	1.50
No formal education	47	11.75
I don’t know	18	4.50
**Time to school**		
Less than 10 min	52	13.00
10–20 min	49	12.25
21–30 min	99	24.75
31–40 min	47	11.75
More than 40 min	153	38.25

This table shows the distribution of respondents as per age, education level as well as mothers’ education and time spent travelling to school.

### 3.2. Menstruation issues for girls

The findings revealed potentially good mother-daughter communication about menstruation followed by peers and teachers, where over half, (60.5%) talked to their mothers followed by friends (21.5%) and teachers accounting for 10.5%. Teachers provided sessions on MHM at school and commonly in separate classes of boys and girls. This prompted girls to ask for more details during sessions as well as with caregivers at home. Persistent social taboos and stigma pose restrictions on activities during menstruation whereby 21.8% were not allowed to prepare food and 17.3% were not allowed to attend religious functions.

**Fig 1:**
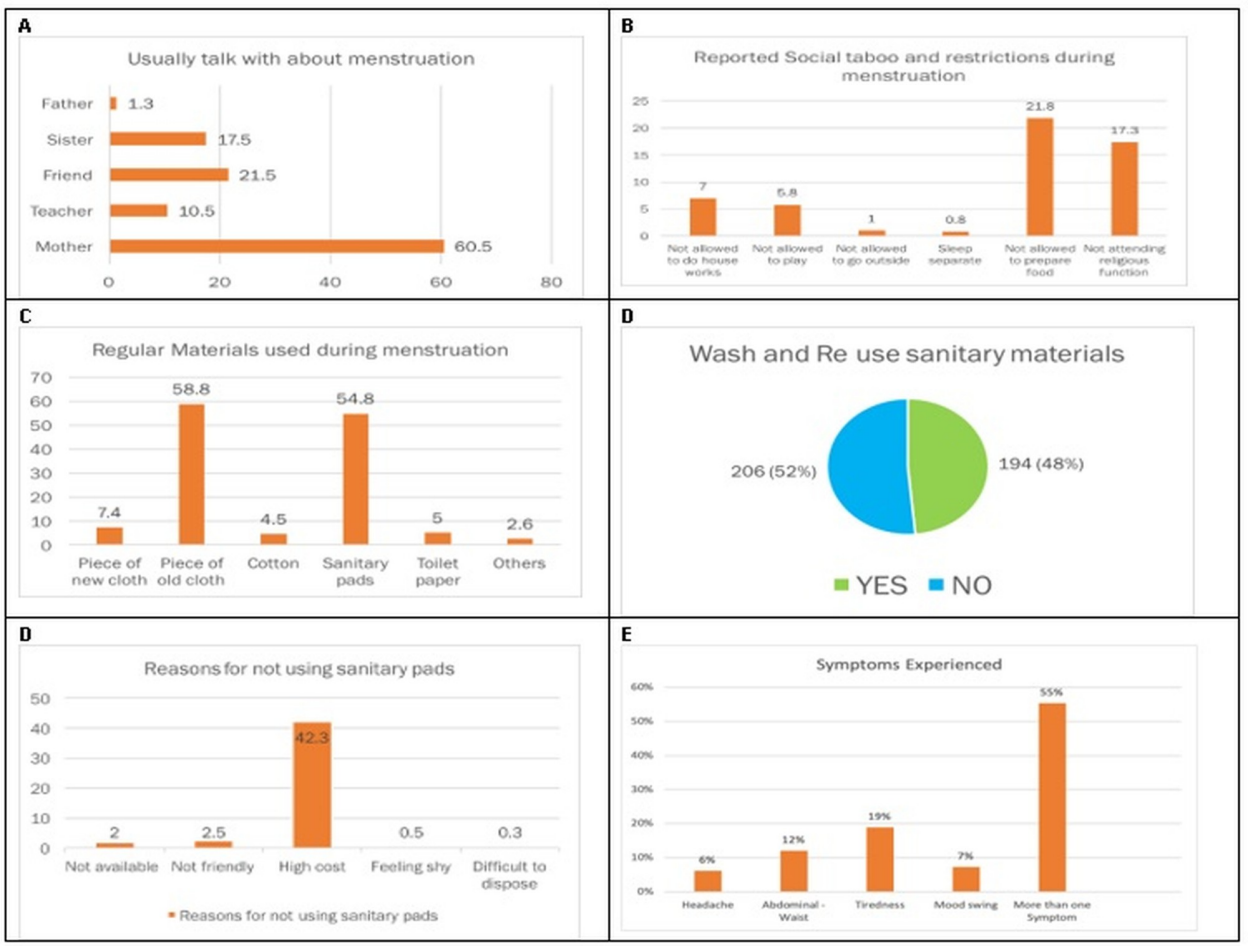
Menstruation issues for girls on social taboos and restrictions, regular material used during menstruation, reasons for not using sanitary pads, and symptoms experienced during menstruation.

On the choices of absorbent materials, 58.8% of girls reported using pieces of old cloth during menstruation this is due to affordability and easy availability and inability to afford non-re-usable sanitary material which was reported by 42.3% of the girls. More than half (52%) preferred the use of reusable sanitary material which was high compared to the use of non-reusable materials which was 48% (Fig 1). They indicated a preference for using reusable materials due to financial constrain since reusable materials save the cost of repeatedly buying non-reusable. Unreliable absorbents keep girls from school, this is the challenge among those using old pieces of cloth particularly the availability of good pieces that can easily absorb. Girls with no absorbent materials reported frequent absences from school and girls reported knowing others who struggled to find absorbent materials and thus stayed home [7].

Abdominal waist pain is the most common physical experience during menstruation along with other symptoms such as headache, and tiredness whereby most girls experience more than one symptom which was reported by 91.5% of the girls. Girls also experience psychological disturbances such as anxiety, and embarrassment resulting from soiling thus leading to absenteeism, especially during heavy flow days 30%, while 33.8% had poor concentration during studies, and 27.5% disengaged during answering questions due to pain in the class. In a study done by Hennegan et al., girls reported worrying about periods because of serious cramps and back pains, and when felt the burden was too much they didn’t go to school [7].

### 3.3. Girls’ menstruation challenges in school setting

**Fig 2:**
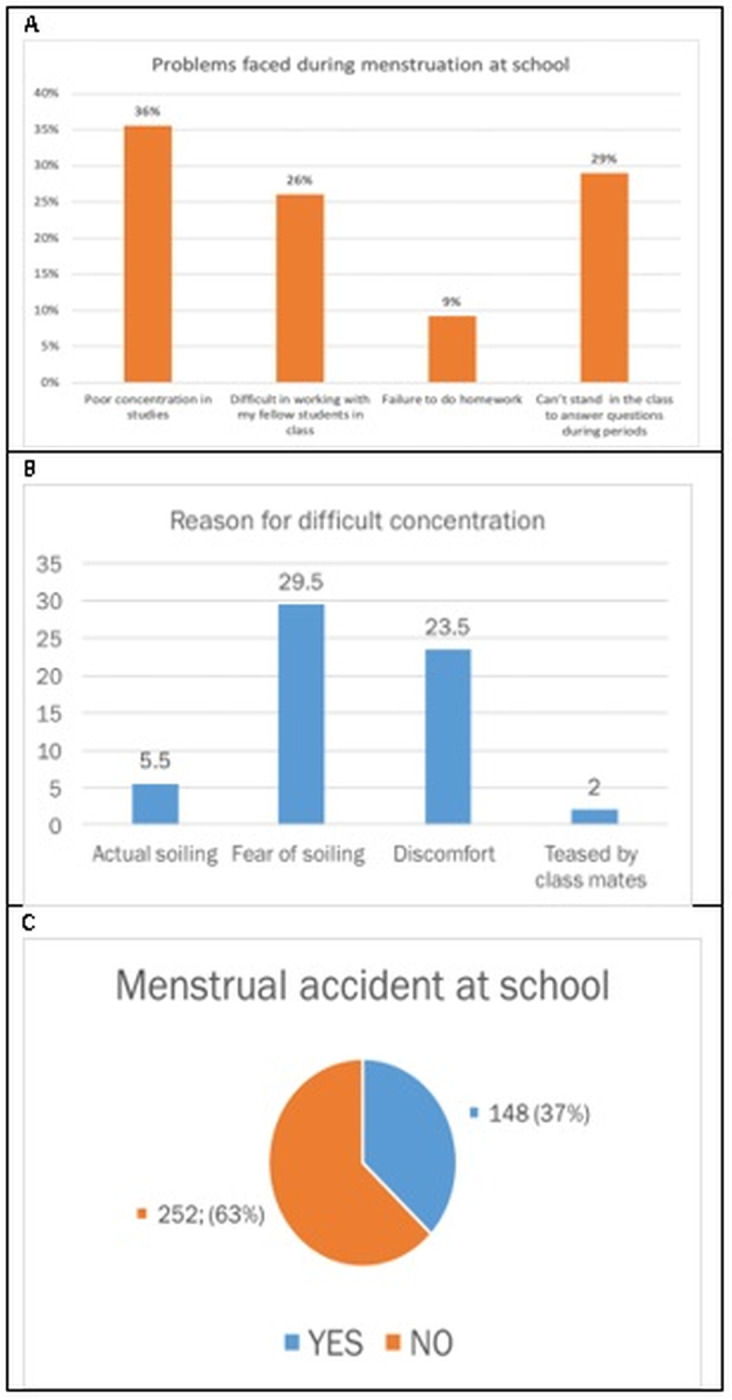
Challenges of girls’ menstruation at school.

About 86% of the students reported problems in class related to menstruation. Fear of soiling and discomfort were the main reasons for difficulty concentrating in class during menses reported by 29.5% and 23.5% of the students respectively (Fig 2). Soiling was reported as a problem, especially by girls who used improper MHM absorbent materials and those with heavy flow, 5.5% reported actual soiling during classes and this limited them to standing up to answer questions in class with 63% reporting experiencing a ‘menstrual accident’ at school. They expressed discomfort and needed to wait to get away from the classroom or school during a break due to fear of showing soiling.

### 3.4. School issues affecting menstruation

**Fig 3:**
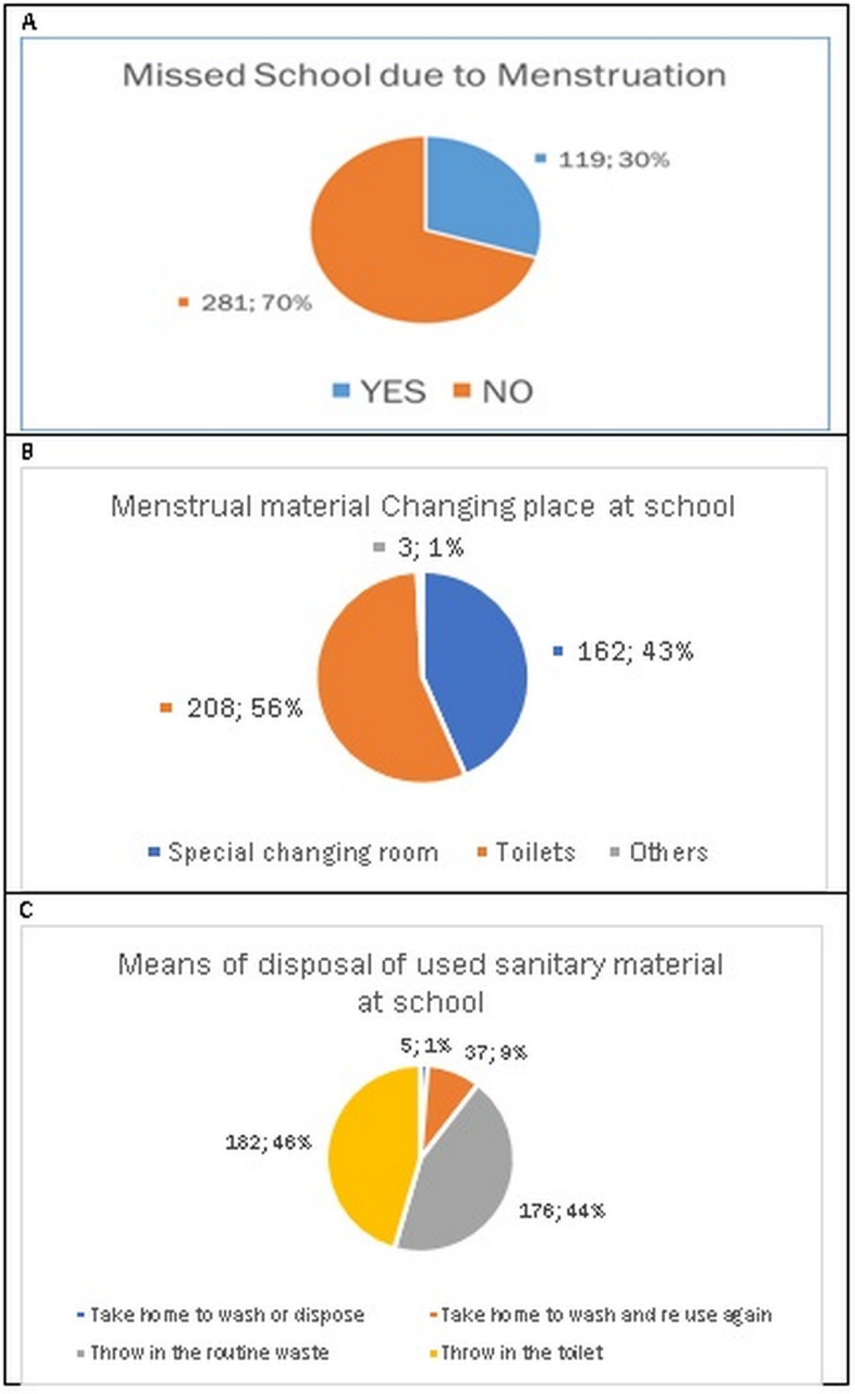
Girls’ menstruation issues at school: Missing school, changing places, and means of disposal of used sanitary materials.

Our study identified multiple factors that inhibit girls’ full participation in school, including inadequate latrine infrastructures, inadequate WASH facilities, unavailability of special changing rooms, and the inability to afford or access sanitary pads. Some girls missed school during menstruation (30%) due to identified significant issues such as the availability of proper washing facilities. For appropriate MHM, both the body and reusable absorbents must be washed several times a day and then dried to meet adequate MHM standards according to current guidelines [24]. Over half (56%) of the girls reported using toilets as a changing place at school (Fig 3). Due to cost barriers, 52% of the girls preferred to use reusable sanitary materials compared to 46% of the students who used sanitary materials in the toilets at school and 44% threw in the routine waste (Fig 3).

### 3.5. MHM practice among girls

**Fig 4:**
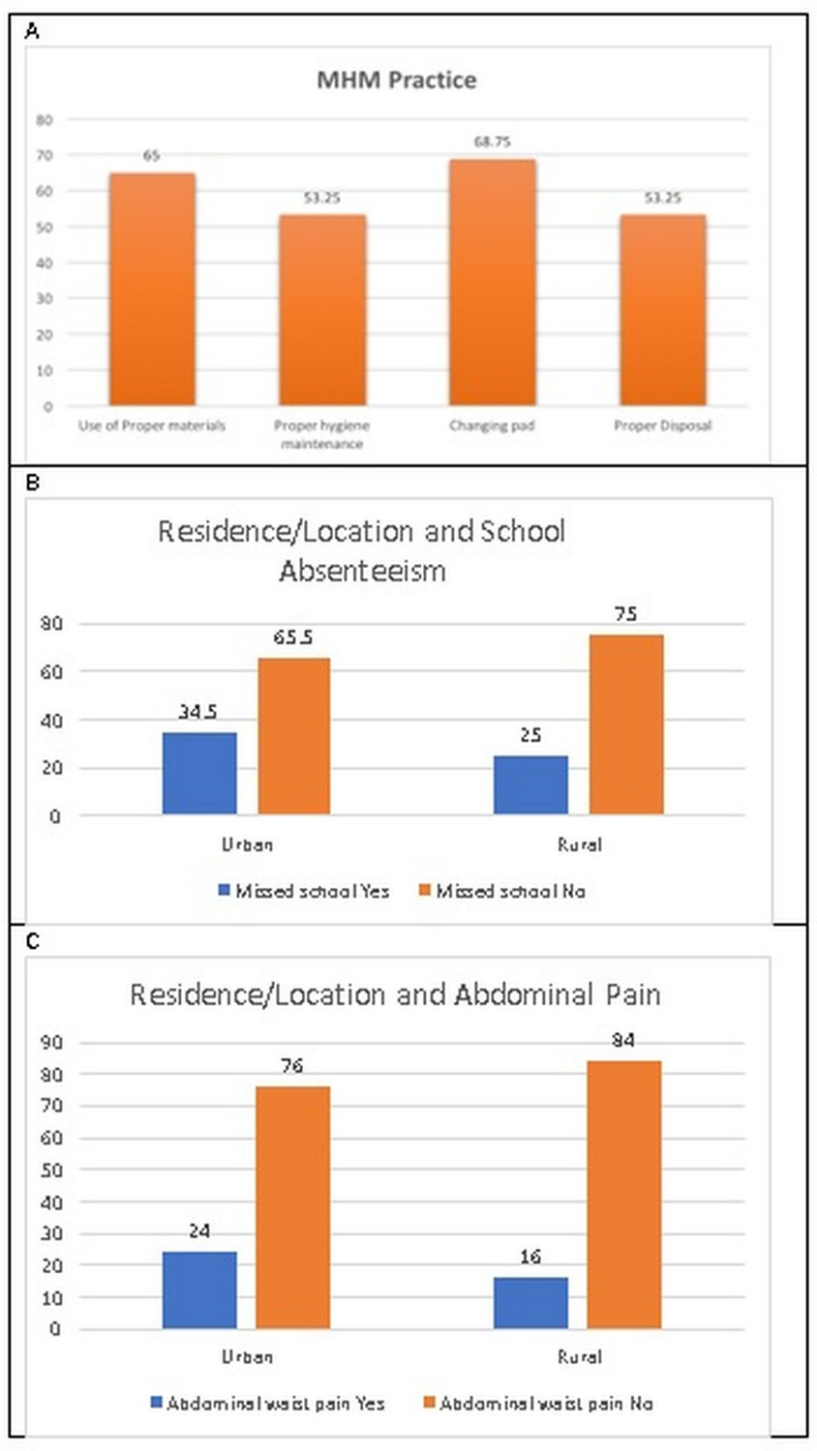
MHM practices among girls.

About 65% of the girls reported proper use of sanitary materials and 53.3% reported proper menstrual hygiene management (In this study proper use and proper MHM was defined as the use of reusable or disposable menstrual materials, change of menstrual materials regular >3 times a day and washing before changing). About 34.5% of students in urban areas missed school during menstruation which is higher compared to those in rural areas which is 25% (*P = 0*.*038*) (Fig 4). In urban areas, 24% of students reported experiencing abdominal waist pain which is higher than students in rural areas who reported abdominal waist pain by 16% (P = 0.046).

### 3.6. Factors associated with school absenteeism during menstruation

Table 2 presents the findings from bivariate and multivariable logistics regression models. The multivariable logistics regression model showed that the odds of missing school during menstruation were three times greater among adolescents presenting with headaches compared to those who do not present with headaches (AOR = 3.29, 95% CI; 1.32–8.23). Also, the odds of missing school during menstruation were eight times greater among adolescents presenting with abdominal waist pain compared to those without such complaints (AOR = 8.50, 95% CI; 6.27–15.56). Furthermore, the odds of missing school were six times greater among adolescents in schools without changing rooms compared to those in schools which having changing rooms (AOR = 5.99, 95% CI; 4.82–7.93).

**Table 2:** Unadjusted and adjusted logistic regression of factors associated with school absenteeism during menstruation.

Variable	Unadjusted OR (95% CI)	*P*-value	Adjusted OR (95% CI)	*P*-value
**Age**				
13–14	1			
15–16	0.76 (0.37–1.56)	0.45		
17–19	0.78 (0.40–1.52)	0.46		
**Education**				
Form I	1			
Form II	0.76 (0.37–1.65)	0.51		
Form III	1.15 (0.61–2.18)	0.66		
Form IV	0.75 (0.83–1.50)	0.42		
Form V	0.92 (0.28–2.95)	0.88		
Form VI	0.92 (0.28–2.95)	0.88		
Sexual Reproductive Health Education				
Not received	1			
Received	0.92 (0.57–1.51)	0.72		
**Mother’s education**				
Primary	1			
Secondary	1.92 (0.73–5.09)	0.73		
College	0.48 (0.55–4.21)	0.509		
University	0.92 (0.46–1.85)	0.46		
No formal education	0.69 (0.22–2.17)	0.52		
**Menstrual Experience**				
Normal	1			
Abnormal	1.62 (0.81–3.22)	0.17		
Headache				
No	1		1	
Yes	3.12 (1.62–6.03)	0.001	3.29 (1.32–8.23)	0.010
Mood				
No	1		1	
Yes	1.65 (0.89–3.08)	0.115	0.46 (0.05–4.44)	0.501
Symptoms				
No	1		1	
Yes	4.79 (1.44–16.00)	0.001	7.18 (1.77–31.56)	0.048
Abdominal waist pain				
No	1		1	
Yes	6.59 (2.29–11.63)	0.001	8.50 (6.27–15.56)	0.001
Tiredness				
No	1			
Yes	1.05 (0.66–1.66)	0.85		
**School Setting**				
Changing Room				
No	1		1	
Yes	4.92 (2.12–9.71)	0.001	5.85 (4.82–7.93)	0.010
Proper material				
No	1			
Yes	1.03 (0.66–1.62)	0.88		
Hygiene maintenance				
No	1			
Yes	0.94 (0.61–1.43)	0.76		
Proper disposal				
No	1		1	
Yes	0.61 (0.39–0.93)	0.024	2.39 (0.32–17.69)	0.393
Changing pads (ref: No)				
No	1			
Yes	1.07 (0.67–1.70)	0.78		

## 4. Discussion

This study highlighted and presented the evidence of challenges faced by adolescent girls in schools. The study emphasized the importance of a supportive learning environment concerning aspects of MHM. While educational curricula address biological aspects of menstruation, girls reported their mother as the primary source of knowledge about menstruation (60.5%) followed by friends (21.5%). Knowledge from teachers was reported at (10.5%). Results show that home teaching on menstrual management is still key for transferring knowledge to girls with the role of school curricula of less importance. The findings reflect those reported in another study conducted in Tanzania [13].

Challenges to a supporting learning environment during menstruation centered on what materials girls used, accessibility of changing spaces, and thus the possibility of appropriate use for either cloth or sanitary pads during the school day. Girls reported using pieces of old cloth regularly were 58.8% while 54.8% reported using sanitary pads regularly. The high cost of sanitary pads was the main reason (42.3%) for not using sanitary pads. In Tanzania at the time of data collection, a tax was introduced on sanitary pads and this increased the cost and many adolescents cannot afford the cost. In a study done in Uganda, girls reported a lack of access to adequate resources, facilities, and accurate information to manage their menstrual hygiene effectively at school [25]. They reported that, as a result, during menstruation, they often struggle at school or miss school [25]. Eighty- six girls (61.7%) reported missing school each month for menstrual-related reasons [5, 9].

While girls reported disposal for used menstrual sanitary materials as 46% in the toilet and 44% in routine waste, the worrying question for the remaining groups, 54% and 56% respectively, would be the likelihood of infrequent changes and possible resulting urinary tract infections. Students who reported using proper materials were 65% and 68.8% change sanitary pads up to three times a day. There was no time breakdown given so that the three times per day could be morning before school, once during or after school, and again before bedtime. Untimely changes could be associated with school absenteeism.

In school settings, the lack of proper places for the disposal of used sanitary pads remains a challenge. The availability of incinerators in schools poses a challenge in disposal as well as positions of incinerators at schools. Growing evidence suggests the gendered impacts of inadequate WASH facilities and support in schools in developing countries influence the participation of girls [7, 14, 20]. Girls have indicated receiving inadequate guidance before their first menstrual period and experiencing fear, shame, and embarrassment in managing menstruation, particularly while in school [14, 20].

Our summary table shows that 30% of the girls reported they missed school due to menstruation. Two factors stand out in the results. First, the lack of changing space in schools is a reported barrier to attendance. Changing spaces need to be safe (with locks on doors, if present) and with access to water and soap. Interestingly, reported barriers of headache and abdominal waist pain were also significant. Analgesics provided by the school could address this issue however school budgets are limited to cover all students in need. Among the girls who participated 33.8% reported poor concentration during studies while they were on menstruation and 27.5% expressed inability/unwillingness for fear of soiling to stand to answer questions during menses in the class.

Another study conducted in Tanzania shows that girls reported difficulty with concentration in classes due to a lack of confidence caused by poor sanitary wear and fear of staining their uniforms, 48% missed class due to menstruation, with 36% staying home during days of heavy flow and 12% not attending school at all during menstruation, 78% responded that menstrual affect academic performance [26].

Addressing the above barriers is vital in ensuring girls have a conducive learning environment during menses. An increasing number of studies have found that girls in low-income settings miss or struggle at school during menstruation if they are unable to manage their menstrual hygiene effectively [9]. In a study done in Bangladesh among schoolgirls who reached menarche, 41% (931) reported missing school, an average of 2.8 missed days per menstrual cycle (8). Students who felt uncomfortable at school during menstruation had a 99% chance of missing school compared to those who didn’t feel uncomfortable, while 64% believed menstrual problems interfere with school performance [20]. Girls recommended having emergency sanitary pads in school which is similar recommendation from a study which was done in Tanzania, Ghana, Ethiopia, and Cambodia [6].

In a study done in Uganda, girls reported a lack of access to adequate resources, facilities, and accurate information to manage their menstrual hygiene effectively at school [25]. They reported that, as a result, during menstruation, they often struggle at school or miss school [25]. In our study, analysis cross-tabulated reported taboos and religion. Taboos exist among girls during menstruation where 21.8% reported not being allowed to prepare food while 17.3% are not allowed to attend religious functions. In these multi-ethnic and multi-religious populations, there was no significant correlation between taboos and ethnicity or religion and missing school during menstruation. This shows physiological factors (abdominal waist Pain) and physical factors like the presence of changing spaces are vital in determining girls’ school attendance during menstruation. In some areas in Tanzania, menstruating girls are not allowed to touch water sources, cook, wash dishes, touch plants, or pass through planted fields [9]. The cultural barriers around menstruation are associated with traditional taboos, such as ideas relating to impurity, witchcraft, and local superstitions, which lead to negative attitudes and practices [9].

Cross-tabulating locations (rural and peri-urban) with school absenteeism showed significant comparative results. Girls in urban schools were 34.5% more likely to be absent from school than girls in rural schools 25%. In addition, girls in urban schools reported significantly higher problems with abdominal pain and discomfort 24% as compared with girls in rural schools 16%.

## 5. Strengths and limitation of this study

The study offers a working example of the quantitative assessment of menstrual hygiene management (MHM), using the best available evidence to assess each aspect. It was conducted by the Council Health Management Team of the Siha Health Department, providing public health practitioners with a hands-on, field experience as a team. Collaboration to organize and conduct a multi-disciplinary study expanded their research experience in the district. Although the project added work time to their regular responsibilities, discussions on the study’s process and results provided insight regarding how to address health issues with a target population in specific settings.

Due to the cross-sectional nature of the study, it is difficult to establish a causal relationship between the dependent and predicting variables. Self-reported menstrual hygiene behaviors, health, education, and psychosocial outcomes are vulnerable to biases, particularly social desirability. Recall bias is a weakness of the study as girls could not accurately recall all days (partial or full days) they missed. They do not keep records of the reasons for missing and cannot openly discuss taboos related to menstruation.

## 6. Conclusion

This study calls for promoting MHM-friendly practices in schools to create a supportive and conducive learning environment for adolescent girls. Ongoing school infrastructure improvements such as the construction of classrooms and toilets in schools should include the construction of proper changing places for adolescents in all schools to reduce the number of adolescent girls who miss school due to lack of a safe, sanitary setting.

We emphasize the importance of having medical emergency kits with essential medications for pain and emergency sanitary pads and the need to reduce tax or have tax-free sanitary pads which will facilitate access to sanitary pads.

The high cost of sanitary pads was found to be a barrier to accessibility among adolescent girls, reducing tax or having tax-free sanitary pads will facilitate access to sanitary pads. It is necessary to improve and ensure the availability of water and soap in changing spaces since those who use and re-use old clothes, without access to soap and water to wash or change the clothes, increase the risk of genital unitary diseases such as Urinary Tract Infections (UTIs).

Focus on urban schools over rural schools referring to barriers and school absenteeism is very important, as the study found the effect of missing schools was significant for peri-urban students.

Continued dissemination of education awareness on MHM to adolescents in schools should be given priority. School health teachers should be trained and supported to increase knowledge and delivery of best MHM practices, facilitating an open exchange between themselves and their students.

## Supporting information

S1 Checklist STROBE statement—checklist of items that should be included in reports of observational studies. (DOCX)

S1 Questionnaire (XLSX)
